# Illinois State Medical Society

**Published:** 1868-07

**Authors:** N. S. Davis, Jos. Robbins

**Affiliations:** Permanent Secretary; Assistant Secretary


### ILLINOIS STATE MEDICAL SOCIETY.

### RECORD OF PROCEEDINGS DURING THE ANNUAL MEETING IN QUINCY, MAY 19TH AND 20TH, 1868.

The members of the Society assembled in Lincoln Hall, in the city of Quincy, and were called to order by the President, Dr. S. W. Noble, at 10½ A.M.

Prayer was made by Rev. Mr. Hunting; and Dr. Bane, in behalf of the Committee of Arrangements, extended to the delegates and members a cordial welcome to the hospitalities of the city of Quincy.

Dr. Jos. Robbins, Secretary of the Committee of Arrangements, reported the names of such delegates and members as had registered. The following is the list of delegates and members in attendance:

*Delegates.*

*Adams County Medical Society.*—Drs. J. W. Bartlett, M. M. Bane, Francis Drude, Julius Gunther, H. W. Kendall, L. H. Baker, N. B. Martin, R. Williams, of Quincy.

*Chicago Medical Society.*—Drs. T. D. Fitch, J. M. Hutchinson, of Chicago.

*Chicago Medical College.*—Drs. N. S. Davis, E. Andrews, of Chicago.

*Fox River Valley Medical Association.*—Dr. D. W. Young, of Aurora.

*Hancock County Medical Society.*—Drs. Geo. W. Hall, of Carthage; A. E. McNeall, Brewsburg.

*Iroquois Co. Medical Society.*—Dr. L. T. Hewins, of Loda.

*Macon Co. Medical Society.*—Drs. S. T. Trowbridge, J. A. W. Hostetler, E. W. Moore, of Decatur.

*Medical Board of Cook Co. Hospital.*—Dr. R. G. Bogue, of Chicago.

*McLean Co. Medical Society.*—Drs. H. Noble, of Heyworth; D. L. Crist, of Bloomington.

*Military Tract Medical Association.*—Drs. S. P. Breed, of Princeton; John M. Morse, H. S. Hurd, of Galesburg; S. R. Crawford, of Monmouth; Benj. Woodward, of Galesburg.

*Morgan Co. Medical Society.*—Drs. C. T. Wilbur, C. J. Lucas, W. S. Edgar, of Jacksonville.

*Quincy Medical Society.*—Drs. P. T. Kinsler, E. D. Helms, S. A. Amorey, P. A. Marks, of Quincy; J. F. McCormick, of Fowler.

*Rush Medical College.*—Dr. Edwin Powell, of Chicago.

*Winnebago Co. Medical Society.*—Dr. A. L. McArthur, of Rockford.

*Permanent Members.*

Dr. J. O. Hamilton, Jerseyville, Jersey Co.

Drs. G. R. Bibb, David Prince, Jacksonville, Morgan Co.

Drs. J. N. Ralston, Louis Watson, P. H. Bailhache, A. Niles, of Quincy, W. M. Landon, of Burton, M. Shepherd, of Payson, Adams Co.

Drs. H. B. Buck, H. C. Barrell, H. H. Roman, Justus Townsend, of Springfield, N. Wright, of Chatham, Sangamon Co.

Dr. S. W. Noble, of Bloomington, McLean Co.

Dr. J. T. Fraser, of Howard's Point, Thos. G. Hickman, R. T. Higgins, of Vandalia, Fayette Co.

Dr. E. P. Cook, of Mendota, LaSalle Co.

Dr. F. O. Earle, Chicago, Cook Co.

The following were also elected permanent members by unanimous vote:

| | | |
|---|---|---|
| J. W. Bonney, of Columbus, | proposed by | Jos. Robbins. |
| John E. Combs, of Bloomington, | " | S. W. Noble. |
| Ed. Holderness, of Weston, | " | " |
| F. A. Warner, of Farmington, | " | S. K. Crawford. |
| Henry M. Hurd, of Galesburg, | " | " |
| R. J. Patterson, of Batavia, | " | D. W. Young. |
| Samuel Milehan, of Camp Point, | " | D. Prince. |

On motion of Dr. M. M. Bane, J. A. Hay, M.D., of La Grange, Mo., and B. J. Bettelheim, M.D., of Brookfield, Mo., were elected members by invitation.

On motion of Dr. L. T. Hewins, of Loda, Dr. T. D. Fitch, of Chicago, was chosen Treasurer *pro tem.*

The Society took a recess of ten minutes, to give the members from each county reoresented an opportunity to select one of their number to constitute a committee for the nomination of officers and the selection of standing committees. When the Society was called to order, the Secretary announced the following as the Nominating Committee:

Adams Co., Dr. W. M. Lander.
Bureau Co., Dr. S. P. Breed.
Cook Co., Dr. R. G. Bogue.
Fayette Co., Dr. R. T. Higgins.

Hancock Co., Dr. G. W. Hall.
Iroquois Co., Dr. L. T. Hewins.
Jersey Co., Dr. J. O. Hamilton.
Kane Co., Dr. D. W. Young.
Macon Co., Dr. J. A. W. Hostetler.
McLean Co., Dr. H. Noble.
Morgan Co., Dr. G. R. Bibb.
Sangamon Co., Dr. H. H. Roman.
Winnebago Co., Dr. A. L. McArthur.
Knox Co., Dr. H. S. Hurd.
Warren Co., Dr. S. K. Crawford.

The committee to which was referred the charges against the Quincy Medical Society, at the last annual meeting, through the Secretary, submitted the following report:

To the Illinois State Medical Society:

Your committee, "appointed to examine and report on the status of the Quincy Medical Society," would, most respectfully, beg leave to report that we have given considerable time and careful consideration to the examination of the statements presented to us by Dr. J. Robbins, on the part of the Adams Co. Medical Society, and of Dr. A. Niles, in behalf of the Quincy Medical Society; and that we can find no just cause for the exclusion of the delegates of the Quincy Medical Society: and would, therefore, recommend the adoption of the following resolution:

*Resolved*, That the Quincy Medical Society is entitled to representation in the Illinois State Medical Society.

C. Goodbrake,

D. O. Crist.

Dr. H. Noble moved that the report of the committee be accepted, and the accompanying resolution adopted; and expressed the hope that the question would be taken without debate.

Dr. Louis Watson objected, and claimed that the whole subject should be discussed.

Dr. J. Robbins was proceeding to discuss the merits of the questions between the Adams Co. Medical Society and the Quincy Medical Society, when Dr. N. S. Davis protested against his proceeding, stating that part of the time of the previous annual meeting had been absorbed on the same topic; it had then been referred to as impartial a committee as could be found in the State; both parties had had a whole year to present the facts before that committee, and he thought the conclusions should be adopted without further waste of time by the Society.

Dr. N. Wright called for the *previous question* on the motion of Dr. H. Noble. The call was sustained by the following vote: Ayes 20, Nays 15.

The resolution reported by the committee was then adopted, by Ayes 19, Nays 12.

The Secretary read a communication from Dr. E. L. Holmes, of Chicago, saying that he has his report partly prepared, but was detained at home by sickness in his family, and requesting that he might have the privilege of giving his report directly "to the Publishing Committee, for publication in the Transactions."

On motion of Dr. T. D. Fitch, the request of Dr. Holmes was granted.

The Permanent Secretary, in behalf of the Publishing Committee, presented the following report, which was adopted by a vote of the Society:

*Report of the Committee of Publication to the Illinois State Medical Society, for 1868.*

As soon after the last meeting of the Society as the papers and reports referred to the committee were received by the Secretary, they were put to press, and 200 copies ordered for the use of the Society. As soon as they were ready, a copy was mailed, postage paid, to every member of the Society who was reported by the Treasurer as having paid his annual assessment for 1867. About 110 copies were thus distributed; about 25 copies were sent to the various medical periodicals, and a smaller number of copies were sent to other medical societies who desired to exchange Transactions with us. Between 40 and 50 copies remain in the hands of the Secretary, for the further use of the Society.

The cost of publishing the Transactions for the year 1867, was $298.61, as shown by the bill of Robert Fergus' Sons, Printers, filed with the vouchers of the Treasurer. The same has been paid in full, together with all other bills against the Society for printing or otherwise. See report of the Treasurer.

All of which is respectfully submitted, in behalf of the Publishing Committee, by

N. S. DAVIS, *Permanent Sec'y.*

The annual report of the Treasurer was presented by the Secretary, as follows:

*Treasurer's Report.*

The Treasurer of the Illinois State Medical Society would respectfully submit his *Sixth* Annual Report. In accordance with the instruction of the Society, he has forwarded a statement of dues to all the members who were not present at the last meeting, and has received, in answer to this correspondence, the sum of $81. The financial statement for the year, ending May, 1868, is as follows:

J. H. Hollister, *Treasurer,*

In acc't with Ill. State Med. Society,

| | | | |
|---|---|---|---|
| 1867. | *Dr.* | | |
| May | To Cash received at the annual meeting at Springfield, as per Treasurer's Record,-- | | $287 00 |
| 1868. Feb. 17. | Am't received by remittance, in answer to correspondence with absent members,---- | | 81 00 |
| | Am't in full,------------------ | | $368 00 |
| | *Cr.* | | |
| | By paid for publishing Transactions and Circulars in full, for 1867,-------------------- | $304 61 | |
| | Expenses of correspondence,----------------- | 3 00 | |
| | Paid for mailing Transactions,-------------- | 7 00 | 314 61 |
| | Balance in Treasury, subject to order,---------- | | $43 39 |

Respectfully submitted,

J. H. HOLLISTER, *Treasurer.*

The report was accepted, and ordered to be printed in the Transactions.

A communication was received from Dr. J. H. Hollister, Chairman of the Committee on Necrology, asking for further time to complete a report, which request was granted by vote of the Society.

The Nominating Committee presented the following report:

*For President*—Dr. S. T. TROWBRIDGE, of Decatur.

*1st Vice-President*—Dr. J. O. HAMILTON, of Jerseyville.

*2d Vice-President*—Dr. J. T. FRAZER, of Howard's Point.

*Treasurer*—Dr. J. H. HOLLISTER, of Chicago.

*Assistant-Secretary*—Dr. T. D. FITCH, of Chicago.

*Place for holding next Annual Meeting*—Chicago.

On motion, the recommendations of the Committee were unanimously adopted.

Drs. L. T. Hewins and E. W. Moore were appointed a committee to conduct the newly elected officers to their places.

Dr. Trowbridge, on taking the chair, expressed his gratitude to the Society for the honor conferred; and Dr. Noble, in retiring, cordially thanked the Society for the uniform kindness and courtesy shown him during the past year.

The Society then adjourned until 1½ o'clock P.M.

*Afternoon Session.*

Society called to order by the President, at 2 o'clock P.M.

Reports from standing committees being in order, were called for as follows:

*On Practical Medicine and Epidemics*, Dr. H. A. Johnson, of Chicago, Chairman. Dr. E. Andrews said he was requested by the chairman of the committee to state that he had been absent in Europe, which, with some degree of ill-health, had prevented his preparing a suitable report.

Dr. E. P. Cook, of Mendota, one of the members of the committee, presented and read a report in relation to the Topography and Diseases of LaSalle County. On motion of Dr. S. W. Noble, the report was accepted and referred to the Committee of Publication.

*On Surgery*, Dr. Edwin Powell, of Chicago, Chairman. Dr. Powell presented an abstract of his report, which related, first, to the treatment of ununited fractures by repeated drillings, as recommended by the late Prof. Brainard; second, to treatment of strictures of the urethra by perineal section; third, cystotomy as a remedy for cystitis; fourth, to the treatment of prolapsus of the rectum; fifth, to the use of carbolic acid in surgery; sixth, to the use of bromine in erysipelas; and seventh, to the resection of nerves for neuralgia.

Dr. E. Andrews moved that the report be accepted and referred to the Committee on Publication. He also commented at some length on that part of the report relating to the resection of nerves, and adduced some cases in his practice, in which operations on the nerve of the lower jaw had proved permanently successful. He advises the operation to be made high enough to embrace the myeloid branch of the nerve.

Dr. W. S. Edgar, in relation to that part of the report on the treatment of ununited fractures, remarked that he regarded the repeated borings with the drill, recommended by Dr. Powell, unnecessary if the *spike* was used properly as an aid. He related one case treated by the *spike* alone, without drilling, and another in which the spike and drill were both used.

Dr. Powell thought the difference between the action of the spike and drill was just the difference between the simple and compound fracture. He claimed that the simple drill was used sub-cutaneously and produced no dangerous degree of inflammation.

Dr. D. Prince remarked, that in the cases in which he had used the *spike* none of the symptoms of compound fracture followed; the spike operating by pressing the fractured parts together more perfectly.

The vote was taken on the motion to refer the report of Dr. Powell, and carried unanimously.

*On Obstetrics*, Dr. E. W. Moore, of Decatur, Chairman. Dr. Moore presented and read an interesting report, which was accepted and referred to the Committee of Publication.

*On Drugs and Medicines*, Dr. Henry Wing, of Collinsville, Chairman. No report, and the committee discharged.

*Special Committees.*

Dr. H. H. Roman, of Springfield, read a report on Ophthalmology, which was accepted and referred to the Committee of Publication.

Dr. N. S. Davis, of Chicago, announced that his report on Cholera was ready, and its hearing was made the special order for 10 o'clock A.M., to-morrow.

Special Committee on the Language of the Pulse in Disease, Dr. J. H. Hollister, of Chicago, Chairman, made no report.

Dr. D. Prince, Special Committee on Fractures of the Lower end of the *Radius*, had not completed his report, and was continued another year.

Dr. E. Ingalls, of Chicago, on Conservatism in the Use of Remedies, made no report.

Dr. R. G. Bogue, of Chicago, Chairman of the Committee on Diseases of the Spine and Joints, read a report on the latter topic, which was discussed by Drs. D. W. Young, E. Andrews, N. S. Davis, and E. Powell. The report was referred to the the Committee of Publication.

On motion, the Society adjourned until 8 o'clock P.M.

*Evening Session.*

The President called the Society to order at 8 o'clock P.M.

Dr. O. F. Earle, of Chicago, member of the Committee on Deformities and Diseases of the Spine and Joints, read a report in relation to the pathology and treatment of deformities of the spine. The report was accepted and referred to the Committee of Publication.

Dr. E. Andrews, of Chicago, read a paper on the Use of the Endoscope, accompanied by an exhibition of the instrument used with a magnesium light. The paper was referred to the Committee of Publication.

The Committee on Nominations reported the following standing and special committees, to report at the next annual meeting of the Society.

*On Surgery*—Drs. A. L. McArthur, of Rockford; D. W. Young, of Aurora; and R. T. Higgins, of Vandalia.

*On Practical Medicine and Epidemics*—Drs. E. P. Cook, of Mendota; D. L. Jewell, of Watseka; and S. P. Breed, of Princeton.

*On Obstetrics*—Drs. H. B. Buck, of Springfield; S. K. Crawford, of Monmouth; and H. B. Cheney, of Joliet.

*On Drugs and Medicines*—Drs. N. S. Davis, of Chicago; Louis Watson, of Quincy; and F. B. Haller, of Vandalia.

*On Gynœcology*—Dr. J. H. Chenowith, of Decatur.

*On Insanity*, to inquire what legislation, if any, is necessary to secure the rights and secure the comforts of the insane, who do not have the benefits of the Illinois Hospital for the Insane—Drs. R. J. Patterson, of Batavia; D. W. Young, of Aurora; and G. R. Bibb, of Jacksonville.

*On Lithotomy*—Dr. E. Powell, of Chicago.

*On Deformities of the Knee and Ankle-Joints, unconnected with Inflammation*—Dr. R. G. Bogue, of Chicago.

*On Ophthalmology*—Drs. E. L. Holmes, of Chicago; H. H. Roman, of Springfield; and J. S. Hildreth, of Chicago.

*On Aphasia*—Dr. H. M. Hurd, of Galesburg.

*On Cholera-Infantum*—Drs. N. Wright, of Chatham, and H. C. Barrell, of Springfield.

*On Idiocy*—Drs. C. T. Wilbur, of Jacksonville; N. S. Davis, of Chicago; H. H. Roman, of Springfield; F. B. Haller, of Vandalia; and S. T. Trowbridge, of Decatur.

*On Staphyloraphy*—Dr. Moses Gunn, of Chicago.

On motion, the report of the Nominating Committee was adopted.

The Society adjourned until 9 o'clock A.M.

SECOND DAY—*Morning Session.*

The Society was called to order at 9 o'clock A.M., Dr. Trowbridge in the chair.

Dr. DeLaskie Miller, of Chicago, special committee on Cholera-Infantum, made no report.

Dr. F. B. Haller, of Vandalia, chairman of the committee to which was referred certain resolutions pertaining to Medical

Education, at the annual meeting of 1867, submitted the following report:

GENTLEMEN OF THE ILLINOIS STATE MEDICAL SOCIETY:

Your committee, to whom was referred the resolution of last year, on Medical Education and Schools, would respectfully report the following preamble and resolutions, *viz.:*

*Whereas,* There is, manifestly, a general desire upon the part of all the medical men of America, to raise the grade of medical education to a higher and more efficient degree; therefore, be it

*Resolved,* That we, the representatives of true medicine of Illinois, in annual meeting assembled, do adopt the resolution on medical schools and education, offered at our last annual meeting.

*Resolved,* That we will receive no young man into our offices to read medicine, unless he is possessed of such qualification as was recommended by the Teachers' Convention of 1867.

*Resolved,* That we should, as exponents of the sentiments of the profession of Illinois, and having the glory of our profession and the welfare of humanity at heart, encourage and patronize such schools only as give the most thorough and systematic course of teaching, *regardless of men or place.*

*Resolved,* That the schools of our own State be requested to so modify and extend their time of teaching as to make them as thorough as the times demand, and equal to any schools in the world. F. B. HALLER, *Chairman.*

Dr. L. Watson moved that the resolutions offered by the committee be adopted.

After some discussion, Dr. H. Noble moved that the resolution offered by Dr. F. B. Haller, at the last annual meeting, be adopted as a substitute for those now reported by the committee.

Dr. D. W. Young moved to amend, by striking out the last clause of the resolution offered last year. The motion was seconded by Dr. Davis, and adopted. Remarks on the subject were made by Drs. Powell, Young, Robbins, Watson, Noble, and Davis, after which the resolution, as amended, was adopted by a *unanimous vote.* The resolution is as follows:

"*Resolved*, That we, the members of the State Medical Society, recommend that the several medical schools in this State adopt the plan of teaching recommended by the recent Teachers' Convention, in Cincinnati, as soon as practicable."

Dr. Jas. Robbins, of Quincy, offered a series of preambles and resolutions, ridiculing the Society for its action in admitting the Quincy Medical Society to representation, which were laid on the table, by a vote of ayes 20, noes 10.

Dr. H. Noble called up and moved the adoption of the following amendment of the Constitution, proposed at the annual meeting of 1867, by Dr. E. W. Moore:

"That Art. 4 of the Constitution be so amended, that the term of office of the President and Vice-Presidents shall commence at the opening of the next annual meeting after their election."

Remarks were made by Drs. W. S. Edgar, S. W. Noble, and T. D. Fitch, in opposition to the proposed change; and by Drs. E. W. Moore, D. W. Young, D. Prince, and A. L. McArthur in its favor. The question being taken, the amendment was adopted by the constitutional majority.

Dr. N. S. Davis, of Chicago, special committee on the Causes, Pathology, and Treatment of Cholera, read a report, which was accepted and referred to the Committee of Publication.

Dr. H. Noble called up and moved the adoption of the following amendment to the Constitution, proposed by Dr. Hollister, in 1867:

*Resolved*, That there shall be a regular standing Committee on Necrology, whose duty it shall be to report, annually, brief biographical notices of deceased members. The same was adopted by the constitutional majority.

Dr. E. Powell, of Chicago, read a volunteer communication, relating to the Treatment of Periodical Fevers by the Hypodermic Injection of Sulphate of Quinine. The communication was accepted and referred to the Committee of Publication.

Dr. D. Prince, of Jacksonville, read a paper on the Operations for Removal of Stones from the Bladder. The paper was accepted and referred to the Committee of Publication.

Dr. T. D. Fitch proposed the following amendments to the Constitution:

1st. Creation of a new standing committee, called the Investigating Committee, or Board of Censors.

2d. An amendment to the paragraph relative to the election of permanent members, in Sec. 2 of the Constitution.

*Amendment First.*—There shall be appointed, annually, a Committee of Investigation, or Board of Censors, consisting of five members, whose duty it shall be to investigate all charges made against any member of this Society, or any organization auxiliary to this Society, and entitled to representation in it; to investigate the status of all applicants for membership in our organization; and perform such other duties as are usually required of such a committee.

*Amendment Second.*—The Permanent Members shall consist of all those who have served in the capacity of delegates, and of such other physicians as may be proposed by two members of this Society, reported on favorably by the Committee of Investigation, and receiving a two-thirds vote of all the members present. They shall be members in good standing of a regularly organized local (city, county, or district) medical society, entitled to representation in this Society, if such society exists in their immediate locality, or within a reasonable distance. Any member losing his membership in his local society shall, by proper evidence being furnished to this Society, forfeit his membership in this organization.

Laid on the table for one year, according to the rules.

Dr. S. T. Trowbridge, of Decatur, Chairman of the Committoo on Legislation, read a report embodying the substance of a law to be submitted to the next Legislature.

Pending the consideration of this report, the Society adjourned until 2 o'clock P.M.

SECOND DAY—*Afternoon Session.*

The Society was called to order by the President, at 2 P.M.

The report of the Chairman of the Committee on Legislation being in order, Dr. W. S. Edgar, in behalf of a majority of the members of the committee, presented and read a second report. After a discussion, in which Drs. Edgar, Niles, McArthur, Young, Robbins, Watson, S. W. Noble, H. Noble, Davis, and Prince participated, the following resolutions were adopted:

*Resolved*, That the Committee on Legislation, which is hereby continued, be instructed to secure, if possible, the passage of an act by the General Assembly, substantially the same as that reported by Dr. H. A. Johnson, for Legalizing Dissections.

*Resolved*, That the Committee on Legislation be instructed to secure the passage of an act by the General Assembly, embracing the main features of the reports of Drs. Trowbridge and Edgar.

On motion of Dr. Prince, Dr. Jayne, of Springfield, was added to the Committee on Legislation.

The following members were duly elected as delegates to the next annual meeting of the American Medical Association:

J. O. Hamilton, of Jerseyville.
D. L. Crist, of Bloomington.
D. W. Young, of Aurora.
H. H. Roman, of Springfield.
A. L. McArthur, of Rockford.
N. Wright, of Chatham.
I. T. Wilson, of Quincy.
S. W. Noble, of Bloomington.
H. S. Hurd, of Galesburg.
J. C. Corpus, of Mendota.
A. Niles, of Quincy.
S. T. Trowbridge, of Decatur.
G. R. Bibb, of Jacksonville.
Peter Young, of Mendon.
T. F. Worrell, of Bloomington.
H. W. Kendall, of Quincy.
T. G. Hickman, of Vandalia.
D. S. Jenks, of Plano.
C. Goodbrake, of Clinton.

Dr. D. Prince offered the following resolution, which was unanimously adopted:

*Resolved*, That the thanks of the Society are extended to the profession and citizens of Quincy, for the adequate provisions made for the exercises of the meeting, and for the hospitality extended to its members.

The Nominating Committee submitted the following report, which was adopted:

*Committee of Arrangements for next Annual Meetiny*—Drs. R. C. Hamill, S. Wickersham, Thos. Bevan, E. Powell, and G. C. Paoli, all of Chicago.

*Standing Committee on Necrology*—Drs. J. H. Hollister, of Chicago; F. B. Haller, of Vandalia; and W. S. Edgar, of Jacksonville.

Dr. D. W. Young offered the following resolution, which was unanimously adopted:

*Resolved*, That the thanks of the Society are hereby tendered to the Chicago, Burlington, and Quincy Railroad Company for their liberality in passing the delegates to this meeting over their road home free of charge.

Dr. Louis Watson expressed much regret and personal feeling on account of the action of the Society relating to the Quincy Medical Society, and asked leave to withdraw his name as a permanent member of this Society. After some remarks by Drs. McArthur, Young, Ralston, and Robbins, on motion, the whole subject was laid on the table.

The Society then adjourned until the third Tuesday in May, 1869.

N. S. DAVIS, *Permanent Secretary.*
JOS. ROBBINS, *Assistant* "

